# Dual spin max pooling convolutional neural network for solar cell crack detection

**DOI:** 10.1038/s41598-023-38177-8

**Published:** 2023-07-09

**Authors:** Sharmarke Hassan, Mahmoud Dhimish

**Affiliations:** Photovoltaics Laboratory, School of Physics, Engineering and Technology, University of York, York, YO10 5DD UK

**Keywords:** Energy science and technology, Renewable energy, Solar energy, Photovoltaics, Solar cells

## Abstract

This paper presents a solar cell crack detection system for use in photovoltaic (PV) assembly units. The system utilizes four different Convolutional Neural Network (CNN) architectures with varying validation accuracy to detect cracks, microcracks, Potential Induced Degradations (PIDs), and shaded areas. The system examines the electroluminescence (EL) image of a solar cell and determines its acceptance or rejection status based on the presence and size of the crack. The proposed system was tested on various solar cells and achieved a high degree of accuracy, with an acceptance rate of up to 99.5%. The system was validated with thermal testing using real-world cases, such as shaded areas and microcracks, which were accurately predicted by the system. The results show that the proposed system is a valuable tool for evaluating the condition of PV cells and can lead to improved efficiency. The study also shows that the proposed CNN model outperforms previous studies and can have significant implications for the PV industry by reducing the number of defective cells and improving the overall efficiency of PV assembly units.

## Introduction

Solar cell crack detection plays a vital role in the photovoltaic (PV) industry, where automated defect detection is becoming increasingly necessary due to the growing production quantities of PV modules and limited application of manual/visual inspection. Previous research has focused on utilizing signal processing and image processing techniques to detect cracks and anomalies in solar cells. However, these conventional approaches often require complex structures and a large amount of data to achieve accurate results.

Convolutional neural networks (CNNs) have emerged as a powerful tool for crack detection, offering several advantages over traditional methods. CNNs can automatically learn and identify patterns in images, enabling them to accurately detect and classify cracks in PV panels, even when the cracks are not clearly visible or have complex shapes. Moreover, CNNs can be trained to detect cracks with high accuracy and efficiency, saving time and resources compared to manual inspection methods. This is especially crucial in the PV industry, where many PV panels need to be regularly and efficiently inspected.

CNNs serve as the dominant deep learning technique and have consistently outperformed most machine-learning approaches in various real-world applications^1,2^. Among the top-of-the-line CNNs, including GoogleNet^3^, ResNet^4^, and DenseNet^5^, the architectures in order to achieve a high level of performance are all professionally designed by experts who have a deep domain understanding due to their experience in both investigating data and the development of CNNs. The problem is that not every user interested in a particular domain is equipped with such domain knowledge. As an example, users who have experience in the data at hand may not necessarily have an understanding of how to build algorithms for CNNs, or vice versa, depending on their familiarity with the data^6^. Therefore, there is a surge of interest in automating CNN architectures, which will make the tuning of CNN architectures transparent to users without any domain knowledge^7–10^. A CNN architecture design algorithm can, on the other hand, promote wide adoption of CNN architectures, thus promoting the development of the field of AI through the development of CNNs.

Based on the type of domain knowledge that is required when implementing the algorithms for CNN architecture design, existing CNN architecture design algorithms can be broken down into two different categories. In the first case, CNN architecture designs are created using a combination of "automatic and manual tuning"^11,12^, and what this means is that manual tuning would still be warranted in addition to the automatic tuning, based on expertise in designing CNN architectures. In this category you will find information about genetic CNN methods and hierarchical representation methods^13^. Another type of CNN architecture design is the so-called "automatic" CNN architecture design^14^, which does not require users to manually adjust its parameters when it is used by them. There is no doubt that the "automatic + manually tuning" design is often superior to the "automated" design when considering the extra benefits that are produced by manual expertise in CNNs^15^. As such, the "automatic" designs have a significant advantage over the "manual" designs, in that they do not need any manual tuning^16^. Users without any domain knowledge of CNNs are much more likely to favour these automated designs.

An innovative deep CNN infrastructure has recently been developed by the authors of^17^ known as Hypotheses-CNN Pooling (HCP), in which an arbitrary number of object segment hypotheses were used as inputs to the system. Each hypothesis was connected to a shared CNN, and finally the CNN outputs for each hypothesis were aggregated with max pooling in order to produce the ultimate multi-label predictions from the CNN outputs. The flexibility of this deep CNN infrastructure can be attributed to a number of unique features, such as the fact that no ground-truth bounding box information is required for training, and that the entire HCP infrastructure is robust to possible noise and/or redundant hypotheses. Another recent study^18^ concluded that degrading an image significantly decreases its classification performance, especially when the training images cannot reflect test image degradation levels. Their visual analysis of the CNN layers revealed that many critical low-level features were not clearly discernible in early layers, which might equate to dropped accuracy.

A recent study^19^ examined the use of CNN networks for medical imaging applications. An experiment evaluated three techniques, including support vector machines with rotation and orientation free features, transfer learning on CNN networks, and capsule network training. Accordingly, CNN methods perform better than traditional methods because they learn and select features automatically. Transfer learning models yield the most accurate results.

Increasing production quantities of PV modules and limited application of manual/visual inspection are driving the need for automated defect detection in the photovoltaic (PV) field. Research in this area has focused on detecting cracks and anomalies in solar cells using signal processing and image processing techniques^20–23^. Nevertheless, a recent study^24^ developed a method for automatically detecting PV module defects in electroluminescence images, using a light convolutional neural network architecture to identify defects in EL images, achieving 93.02% accuracy on the solar cell dataset. Additionally, the classifications of solar cell defects are based on two machine learning approaches proposed by^25^ utilizing features extraction-based support vector machines (SVMs) and convolutional neural networks (CNNs). Using suitable hyperparameters, algorithm optimizers, and loss functions, they have achieved 91.58% accuracy in cell detection classification.

It takes a large amount of data to compile the existing CNN-based solar cell detection methods (usually more than 100 images for each cell) as well as highly complex structures for the CNN to work accurately^26,27^. In recent years, CNN-based algorithms for the detection of solar cell cracks have also been tested in non-industrial settings, where they gain access to EL images and develop their models afterwards, without purifying the validity of their models when applied in an industrial setting (for example, time of processing, speed of cracked solar cell detection, detection of abnormal solar cell structures, test the CNN model against different solar cells containing different busbars, etc.).

Typically, CNN models are slow due to an operation known as “max pooling” within their architecture^28^, and this method is frequently used to inspect solar cells. In real-world applications of CNN models, training CNNs with multiple layers takes a long time if a computer does not have a high-performance GPU. Neither max pooling nor multi-layer CNNs were used in our work.

CNN are becoming increasingly important in the PV industry for crack detection. The use of CNNs in crack detection can provide several benefits over traditional methods. One of the main benefits of using CNNs for crack detection is their ability to automatically learn and identify patterns in images. This allows them to accurately detect and classify cracks in PV panels, even in cases where the cracks are not clearly visible or have complex shapes. Additionally, CNNs can be trained to detect cracks in images with high accuracy and efficiency, which can save time and resources compared to manual inspection methods. This is particularly important in the PV industry where large number of PV panels need to be inspected regularly and efficiently. Moreover, the use of CNNs for crack detection can improve the safety and reliability of PV systems by detecting and preventing cracks that can lead to system failures or safety hazards. This can help to increase the overall performance and lifespan of PV systems, which can lead to cost savings for PV system owners and operators.

Our work expands upon the existing approaches in the field of CNNs for crack detection, aiming to enhance the detection ability in this domain. Through the development of a CNN architecture specifically tailored for crack detection, we have introduced new elements to improve the effectiveness of the model. By employing a training network, we conducted thorough evaluations of different architectures and made necessary adjustments to enhance validation accuracy. Notably, our modifications included transitioning from mean pooling to max pooling, increasing the number of convolutional layers, and introducing a novel pooling method. These enhancements resulted in a significant improvement in validation accuracy, achieving a noteworthy level of 96.97%. To highlight the distinctiveness of our approach, we named it "DSMP-CNN" denoting the utilization of a Dual Spin Max Pooling Convolutional Neural Network architecture. The DSMP-CNN architecture represents a unique variation of CNNs that incorporates the dual spin pooling mechanism along with the conventional max pooling layer. This innovative approach is strategically designed to enhance the accuracy of crack detection by capturing the distinctive features of cracks and other defects present on solar cells. By integrating the DSMP-CNN architecture into our proposed system, we have achieved highly precise evaluations of the acceptance/rejection status of photovoltaic cells, based on the identification of black spots, cracks, PIDs, and shaded areas.

In summary, the major contributions of our research can be highlighted as follows:

- Novel CNN Architecture: We propose the DSMP-CNN architecture, which combines the advantages of mean pooling and max pooling with a dual spin pooling mechanism. This unique approach captures distinctive features of cracks and other defects on solar cells, leading to improved accuracy in crack detection.
- High Accuracy and Efficiency: Through extensive testing on various solar cells, our proposed DSMP-CNN architecture achieves a high degree of accuracy, with an acceptance rate of up to 99.5%. This demonstrates the effectiveness of our approach in accurately evaluating the acceptance/rejection status of photovoltaic cells based on the presence and size of defects, including cracks, microcracks, PIDs, and shaded areas.
- Real-World Validation: We validate the performance of our system using real-world cases, such as shaded areas and microcracks, which are accurately predicted by the DSMP-CNN architecture. This validation showcases the robustness and reliability of our approach in practical scenarios.
- Improved Efficiency and Reduced Defective Cells: By leveraging the power of DSMP-CNN, our research has significant implications for the PV industry. The proposed system can reduce the number of defective cells by accurately detecting and preventing cracks and other defects. This leads to improved efficiency and overall performance of PV assembly units, resulting in cost savings for PV system owners and operators.

## Materials and methods

**Figure 1:**
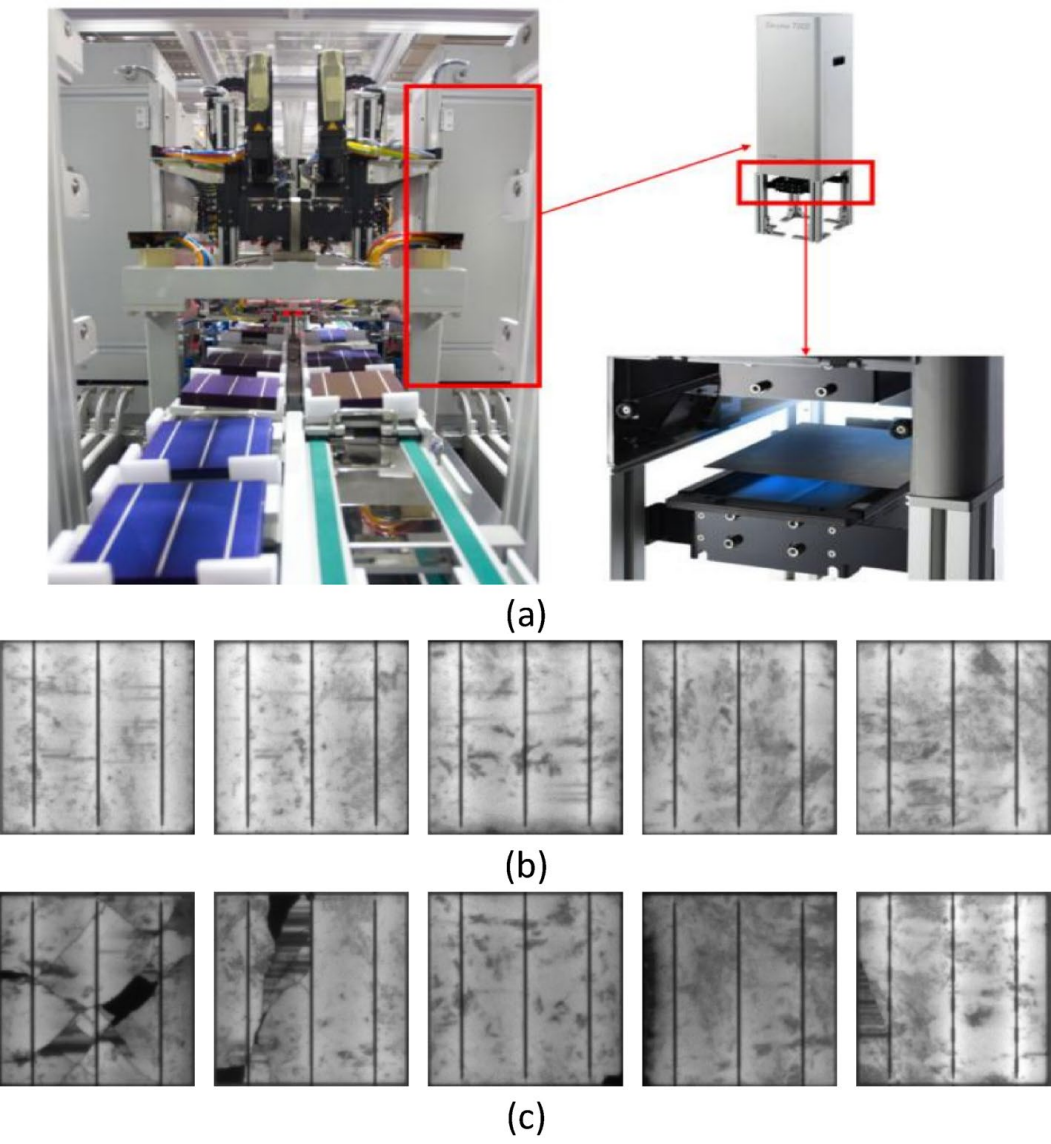
(**a**) Solar cell production line with in-house EL detection equipment, (**b**) Example of solar cells without any cracks “healthy samples”, (**c**) Example of solar cells with cracks “cracked samples”.

The CNN network is implemented using EL images taken directly from a manufacturing solar cell line facility, as shown in Fig. 1a. PV cells can be tested under EL cameras to find hidden defects in their structure; however, as described earlier, automation of detection is necessary for fast decision-making about whether the solar cell is cracked. In Fig. 1b, five healthy (non-defective) solar cells are displayed, while defective solar cells are shown in Fig. 1c. We implemented the CNN as part of the EL setup, so that the process could automatically determine if a solar cell should continue in production (defect-free cells) or if it should be reported as damaged and go into the recycling process (defective solar cells).

EL imaging is a technique used to visualize the electrical activity within a solar cell. In this process, a high-voltage electrical current is applied to the solar cell, causing it to emit light. This light can be captured using a camera, and the resulting image can be analyzed to gain insight into the performance of the solar cell. EL imaging can be used to identify areas of the solar cell that are not functioning properly, such as regions with high resistance or low light absorption (Fig. 1c). Additionally, it can be used to optimize the design of the solar cell to improve its overall efficiency.

In this study, the EL image resolution employed for CNN training, validation, and testing purposes ranges from 1000 × 1000 pixels to 2500 × 2500 pixels. This deliberate variation in image resolution was implemented to ensure that the developed detection algorithm can handle a wide range of resolution levels, encompassing both low and high-resolution images. By incorporating diverse resolution images, the algorithm's effectiveness and robustness across different image qualities are thoroughly assessed and validated in this work.

When building CNNs, the following layers are typically utilized:

- The core of a CNN is the **convolution layer**. This layer contains a set of filters (or kernels), whose parameters are learned throughout the training process. A filter usually has a smaller size than the actual image. An activation map is created by combining each filter with an image. Each dot product is calculated at each spatial position between every filter element and the input when the filter is sliding across the image height and width.
- **Batch normalization, or batch norm**, is the second layer of CNN. During training, it ensures regularization, prevents overfitting, and increases the speed of CNNs. In this layer, each feature map is normalized along with its parameters. The batch norm would result in each feature having a different mean and standard deviation, and therefore, the CNN will produce additional accuracy in image feature extraction.
- In CNN, the third layer is referred to as the **rectified linear unit (ReLU)**. This layer removes all negative values from the filtered image and replaces them with zeros. A certain quantity must be present as input for this function to activate. In other words, if the input is below zero, the output will also be zero. There is, however, a linear relationship between the input and the dependent variable once the input reaches a certain threshold. This means that it is capable of accelerating a deep neural network's training data set at a faster rate than other activation functions.
- **Pooling** is the fourth layer of the CNN. Pooling can be divided into two types: max Pooling and mean Pooling. By using Max Pooling, we are able to extract the maximum value from a specific portion of an image covered by the kernel. Meanwhile, mean pooling is a method for averaging the values from a portion of the image which is covered by the kernel. While in practice, when it comes to selecting whether to use maximum or mean pooling, there is no standardised solution, so the choice needs to be made while training and verifying the accuracy of the CNN network over time.
- A **fully connected layer** refers to a neural network that comprises neurons that apply a linear transformation to input vectors in order to find a solution to the problem through the use of weight matrices. Consequently, every possible connection between the input vector and the output vector is present as a result of the use of layers, such that every input of the input vector influences every output of the output vector.
- In CNN network that predict a probabilistic distribution based on a multinomial distribution, the **softmax function** is used as the activation function in the output layer of the neural network model. This means that softmax is used as the activation function in multi-class classification problems when more than two class labels are required for a class to have membership in the CNN classification.
- In the CNN network, the last layer is referred to as the **classification layer**. This is where a neural network is able to classify a class according to the rules that are defined by the CNN network. Two classes are included in our work, accept and reject, to indicate the status of the solar cell. In the event of cracks in the image, the solar cell will be rejected and will be transferred to the recycling unit of the PV manufacturing unit; otherwise, if CNN accepts the image, the actual solar cell will be placed in the manufacturing assembly unit.

**Figure 2:**
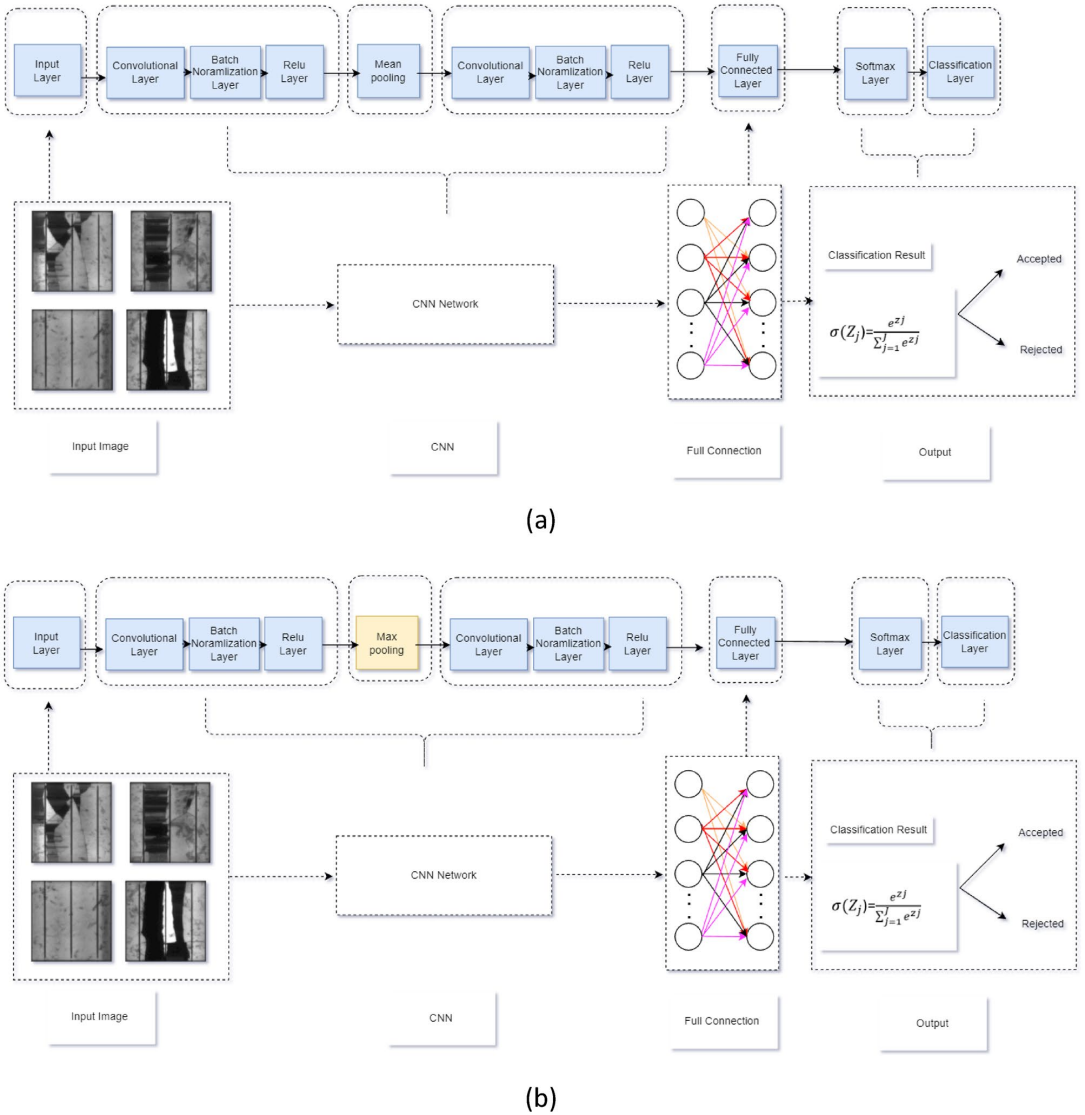
Developed CNN network architectures. (**a**) Two-layer convolutions and mean pooling (referred as Net1), (**b**) Two-layer convolutions and max pooling (referred as Net2).

To begin developing a CNN architecture for crack detection, we began by creating a training network from scratch. The architecture of the training network is divided into several stages, including input, convolutional, pooling, fully connected layers, and output. The initial convolutional network, referred to as Net1/Net2, started with an input layer of resized images of 227 × 227× 3 pixels. This was followed by two layers of convolutional filters with 32 filters, each connected with a batch normalization layer and a Relu activation layer. Mean/max pooling was applied, as illustrated in Fig. 2a and b. During initial experiments, the learning rate was set to 0.0001, the mini-batch size was set to 16, and the network was trained for 20 epochs. A summary of the key parameters of the architecture can be found in Table 1.

**Table 1:** Summary of CNN input parameters for Net4.

Parameter	Value	Parameter	Value
Image input size	227 × 227x3 Pixels	Initial learn rate	0.0001
Convolutional layers	32 filters	Epochs	20
Filter size	3, 3	Mini batch size	16
Learn rate drop factor	0.1	Validation frequency	16
Random rotation (degree)	− 90, 90	Solver	Sgdm

Using the training network, it was determined that the validation accuracy of Net1 is 81.5%, which is the standard for mean pooling, which is quite far from its validity. The accuracy improved to 87.5% when the mean pooling was repleted to max pooling, which is still not significant, but it is making progress towards the validity.

**Figure 3:**
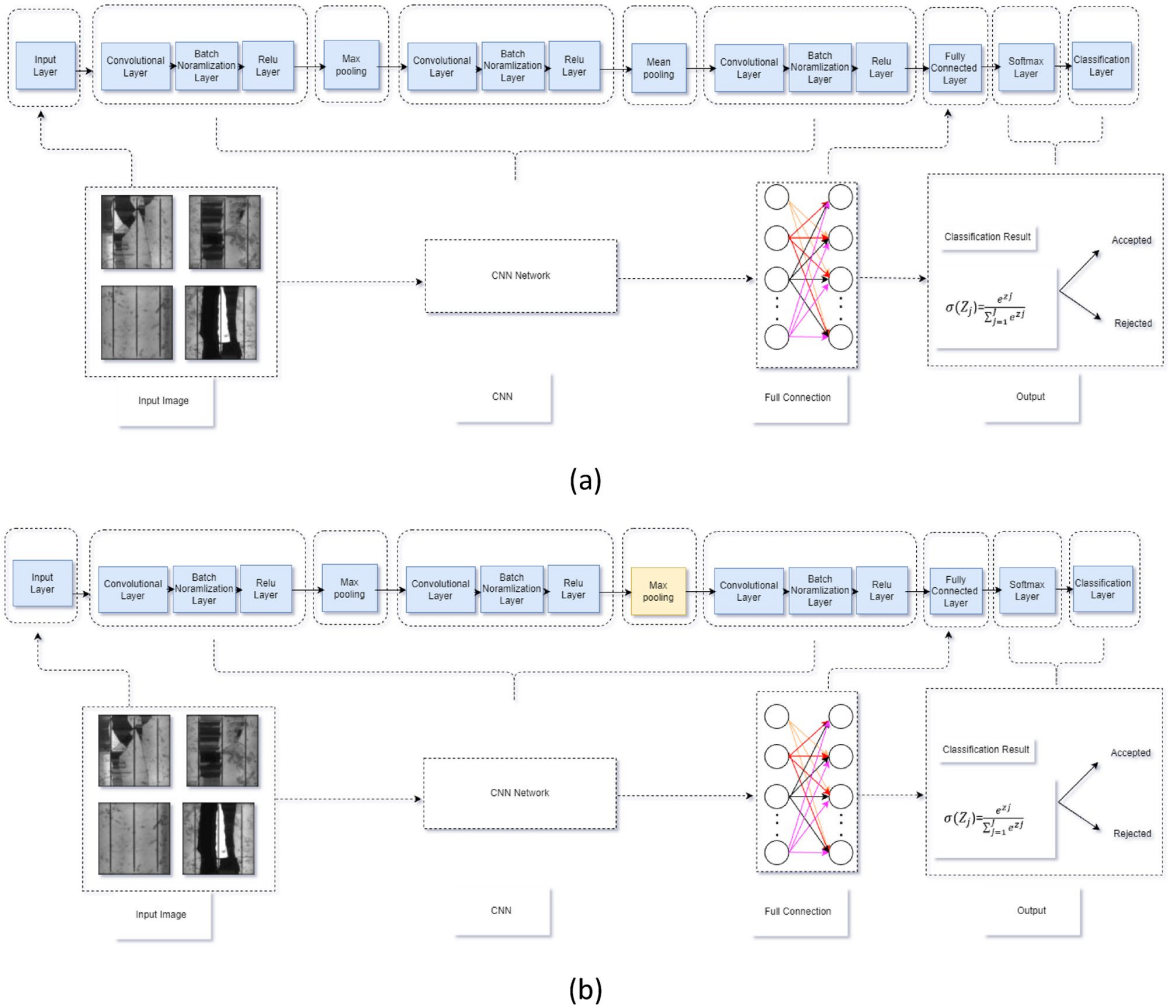
Enhanced CNN network architectures. (**a**) Three-layer convolutions and max-mean pooling (referred as Net3), (**b**) Three-layer convolutions and max-max pooling (referred as Net4).

Following that, the main task was how to improve the validity accuracy of the architecture. In this case, we increased the number of convolutional layers from two to three by using double pooling maximum and mean, as shown in Fig. 3a, and keeping all the other parameters and learning factors unchanged. From there, we were able to improve the validity accuracy to 93.75%.Taking into consideration the fact that the three convolution layers improved the validation accuracy, we decided to keep the layers the same but changed the design of the architecture, changing the double pooling from Max and mean to double Max pooling as shown in Fig. 3b. From there, the validation accuracy improved to 96.97%, which demonstrates that Net4 is the most appropirate architecture to employ for our research.

**Figure 4:**
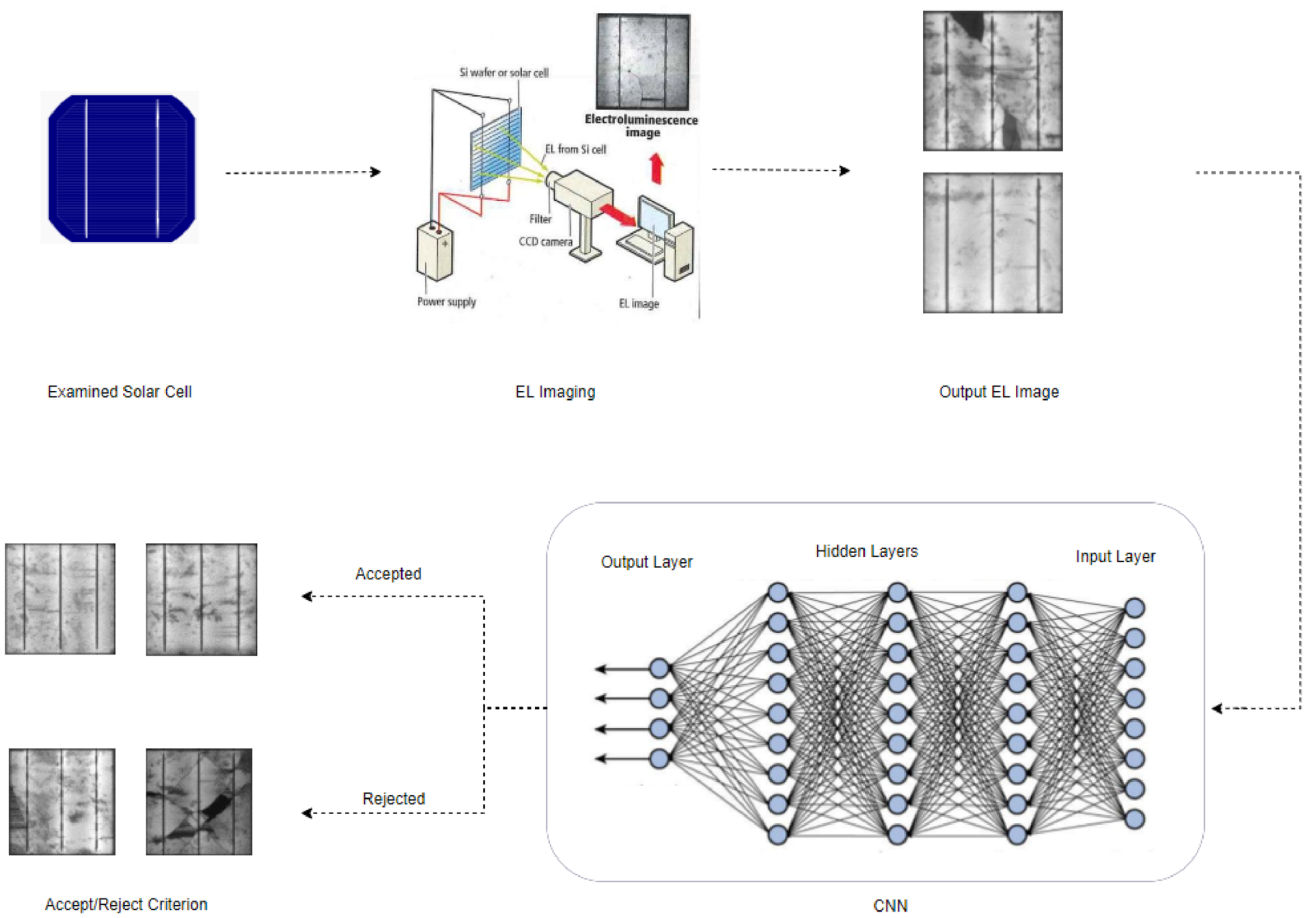
Incorporating the CNN network into decision making for the identification of solar cell cracks in an industrial setting application.

As shown in Fig. 4, the deployment of a CNN network for solar cell inspection begins by capturing an EL image of the solar cell as it exits the manufacturing line. EL imaging is a method used to test the quality of a solar cell by measuring the light emitted from the cell when a small current is applied to it. Once an EL image of the solar cell has been obtained, it is then incorporated into the CNN network for analysis. The CNN network is trained to recognize patterns and features in the image that indicate the presence of cracks or fractures in the solar cell. It uses this information to decide whether to accept or reject the cell.

If the CNN network determines that the cell is free of cracks or fractures, it is classified as "accepted" and sent for assembly into a solar panel. On the other hand, if the network detects cracks or fractures in the cell, it is classified as "rejected" and sent for recycling. The use of a CNN network in this process allows for a high level of automation and accuracy in the inspection of solar cells, as the network can quickly and reliably identify defects that may not be visible to the human eye.

**Figure 5:**
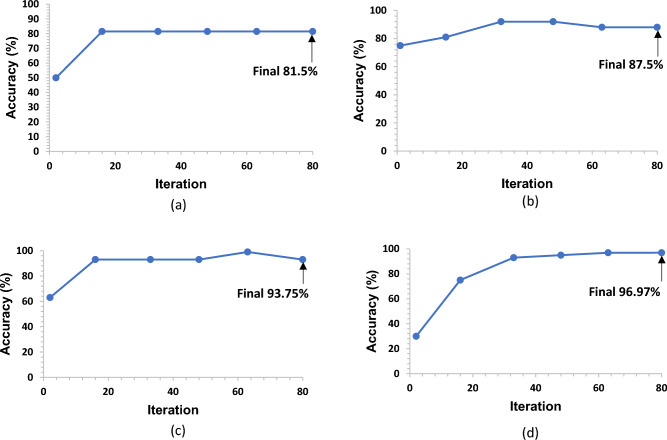
Validation accuracy of CNN network architectures. (**a**) Net1, (**b**) Net2, (**c**) Net3, (**d**) Net4.

During the development of the CNN architecture for solar cell inspection, one of the major challenges faced was adjusting parameters that affect the accuracy, such as the number of training cycles (epochs), validation accuracy, and the rate at which the model learns (learning rate). It was discovered that if the learning rate was too high, the model would converge to a suboptimal solution quickly, and if the learning rate was too low, the process could become stuck^29,30^. To overcome this challenge, the team started by using a learning rate of 0.01 and 10 epochs for the first CNN network (Net1). Initially, the validation accuracy was 56%, but this was gradually improved by increasing the learning rate to 0.0001 and increasing the number of epochs to 20. This resulted in a maximum validation accuracy of 81.5% for Net1 as seen in Fig. 5a.

Since further increasing the number of epochs was not improving accuracy, the team focused on improving the CNN architecture. They replicated the mean pooling of Net1 to the maximum pooling of Net2, which resulted in an improvement in validation accuracy of 87.5%, as seen in Fig. 5b. We continued to improve the architecture by keeping the same learning rate and epochs and adding three convolution layers with max-mean pooling and max-max pooling, respectively, resulting in validation accuracies of 93.75 and 96.97% for Net3 and Net4 as seen in Fig. 5c and d. This allowed them to achieve a high level of accuracy with 20 epochs and a learning rate of 0.0001.

Similarly, the loss function for CNN models is a key parameter to consider, as it measures the variance between the predicted output and the actual ground truth data. To accomplish this, all the critical parameters of the CNN model will be adjusted through the training process with the objective to minimize the loss function in a way that will enhance the model's ability to predict the loss function more accurately as well as its overall performance.

**Figure 6:**
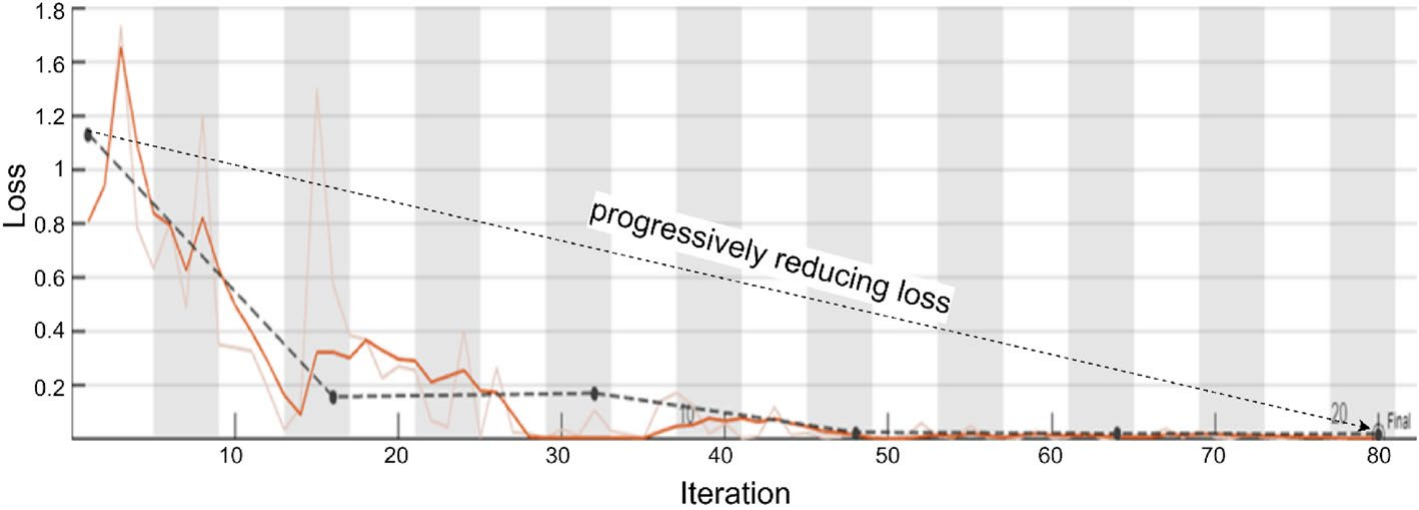
Net4 CNN network learning Loss vs learning iterations (epochs).

An ideal loss graph has two lines labelled red for training loss and blue for validation loss, both of which need to decrease and converge to indicate that the loss model is reducing the prediction error of the model. Based on the Net4 Architecture, shown in Fig. 6, it appears that initially, the loss of the model was a fraction higher, but as the model was constantly trained, the loss of the model was progressively reduced towards zero, showing a high degree of learning and that the model is performing well and minimizing both loss and error.

A confusion matrix is a table that summarizes the performance of a classification model by displaying the number of correct and incorrect predictions made on a dataset. In this case, Table 2 presents the results of the confusion matrix for the CNN model Net4, which was applied to 150 images consisting of 75 healthy solar cells and 75 cracked solar cells. Additionally, the accuracy and precision of the model were calculated using (1) and (2), and the results are as follows:

- The accuracy of 93.3% indicates that the model correctly classified 93.3% of all the samples, encompassing both cracked and non-cracked solar cells in the dataset. This means that the model's overall performance in accurately predicting the class of the solar cells was 93.3%.
- The precision of 92.2% implies that out of all the samples the model identified as cracked, 92.2% of them were indeed cracked solar cell images. Precision measures the proportion of correctly predicted positive cases, indicating that the model's ability to correctly identify cracked solar cells was 92.2% precise.

**Table 2:** Confusion matrix of the developed CNN model “Net4”.

		Actual value	
		Actual no cracks	Actual cracks
Predicted value	Predicted no cracks	**71**	*4*
	Predicted cracks	*6*	**69**

Significant values are in bold and italic.

These results suggest that the CNN model Net4 performed well in classifying the solar cell images, with a high accuracy and precision. However, it's important to note that the interpretation of these results relies on the assumption that the values in the confusion matrix are accurate and correctly reported.

1 $$Accuracy=\frac{TP+TN}{TP+TN+FP+FN}=\frac{71+69}{71+69+6+4}=93.3\%$$

2 $$Precision =\frac{TP}{TP+FP}=\frac{71}{71+6}=92.2\%$$

## Results and discussion

**Figure 7:**
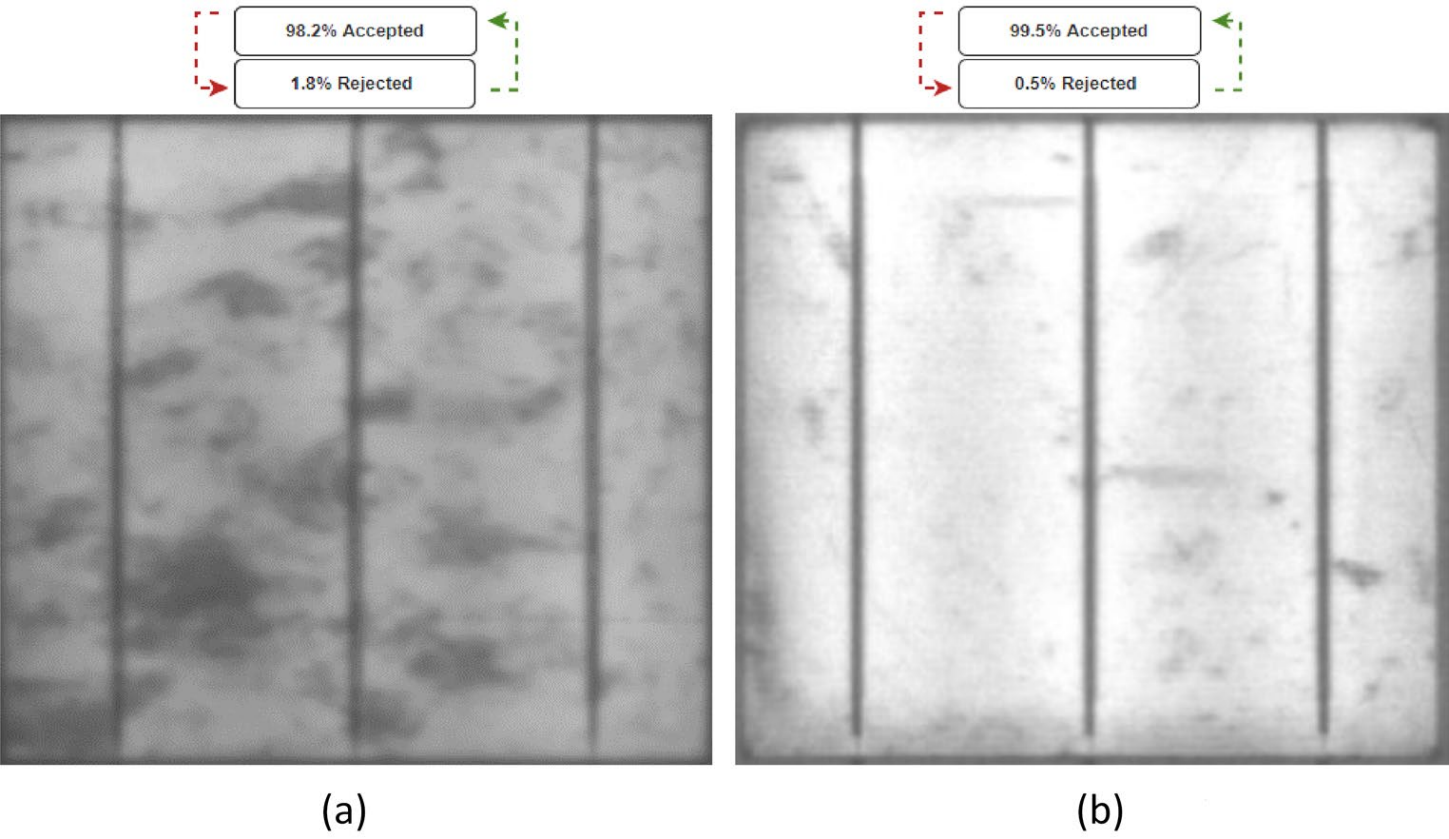
Examined healthy solar cells with an acceptance/rejection percentage. (**a**) Case 1, (**b**) Case 2.

The proposed CNN was tested by inserting various solar cells into the system and evaluating the accuracy of its predictions in determining whether the solar cell should be accepted or rejected. The first solar cell examined was a relatively healthy one with minor black spots (these usually appear in the EL due to the resolution/calibration of the camera), as shown in Fig. 7a. To ensure consistency and facilitate effective learning, the captured EL images were pre-processed to ensure a uniform resolution or resized to fit a specific resolution range (e.g. 1000 × 1000 pixels to 2500 × 2500 pixels). The system predicted that the cell would be accepted with 98.2% accuracy and rejected with 1.8% accuracy, which is a precise prediction as the cell is in good condition and has only a small number of black spots. The system made this prediction by analyzing the size, shape, and quantity of the black spots on the solar cell and comparing them to a database of accepted and rejected solar cells, using mathematical algorithms to predict the cell's acceptance/rejection status with a high degree of accuracy.

The second case (Case 2) was a healthy solar cell with fewer black spots (better EL image resolution) than the first case, as shown in Fig. 7b. The model predicted that the cell had a 99.5% acceptance rate and a 0.5% rejection rate, resulting in accepting the cell for the assembly phase. This illustrates that the proposed model is more precise in predicting the performance of a solar cell than conventional methods, and that it can recognize cells with fewer black spots, which can lead to improved efficiency.

**Figure 8:**
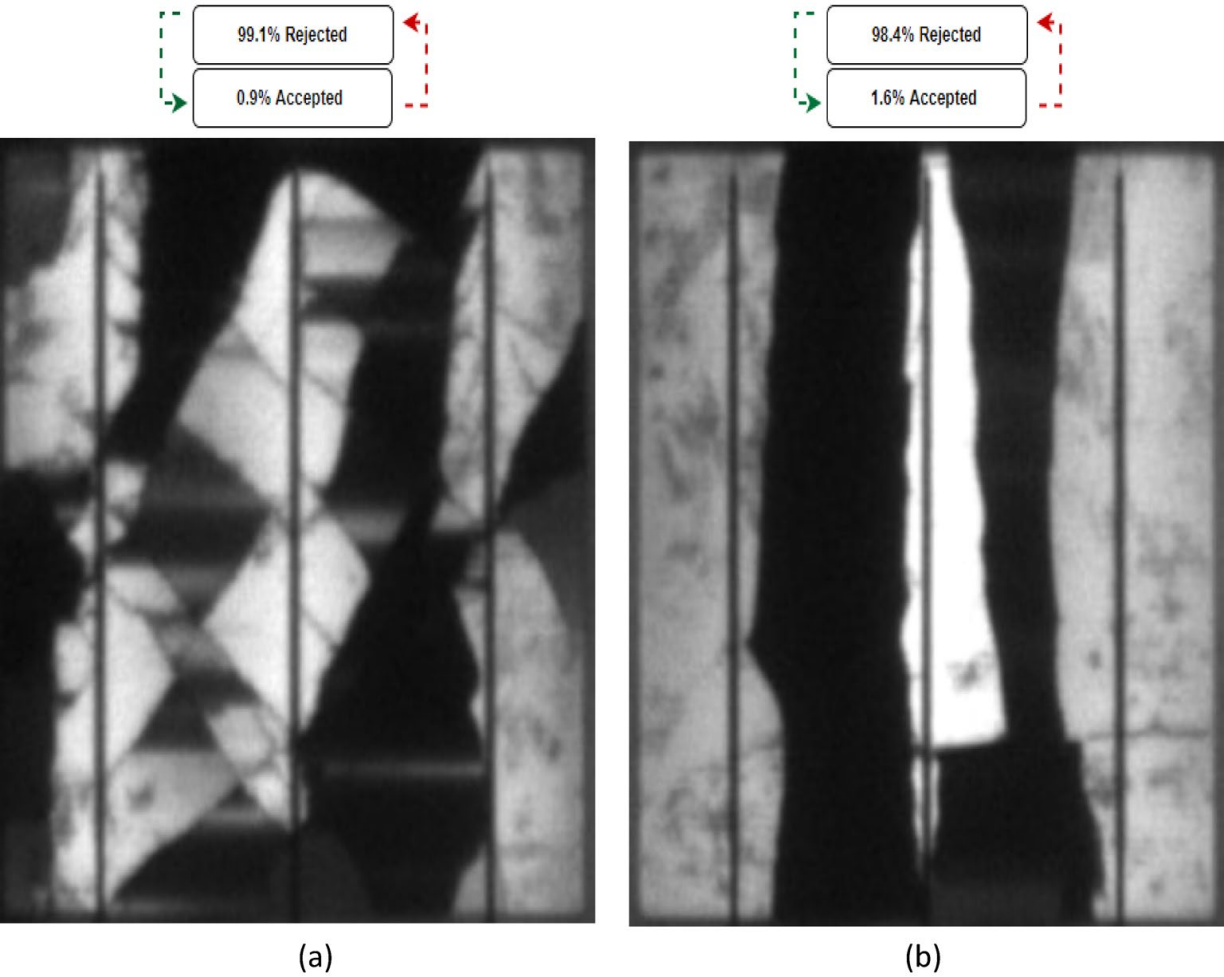
Examined cracked solar cells with an acceptance/rejection percentage. (**a**) Case 3, (**b**) Case 4.

In a subsequent phase of experimentation, solar cells with significant cracks were analyzed. Specifically, Case 3, as depicted in Fig. 8a, focused on the examination of solar cells with major cracks. Upon running the system, it was determined that the solar cell had a rejection rate of 99.1% and an acceptance rate of 0.9%, which is a valid prediction as the cell is visibly damaged by the presence of significant cracks.

To further validate the reliability of the testing, an additional examination of cracked solar cells was conducted. This was done on Case 4, which was similar to Case 3, but with fewer cracks present, as shown in Fig. 8b. The system's prediction was that 98.4% of the solar cells would be rejected, which is still a reasonable estimate, but the rejection rate was lower than that of Case 3, due to the presence of fewer cracks. A further example of crack detection can be found in Figs. S1 and S2 for 12 different healthy and cracked solar cells samples, respectively.

Based on the analysis of four distinct cases, it appears that the proposed system is capable of providing accurate predictions, as evidenced by the reference images. However, it should be noted that certain cases may present more of a challenge to predict, and these will be discussed in the following section.

**Figure 9:**
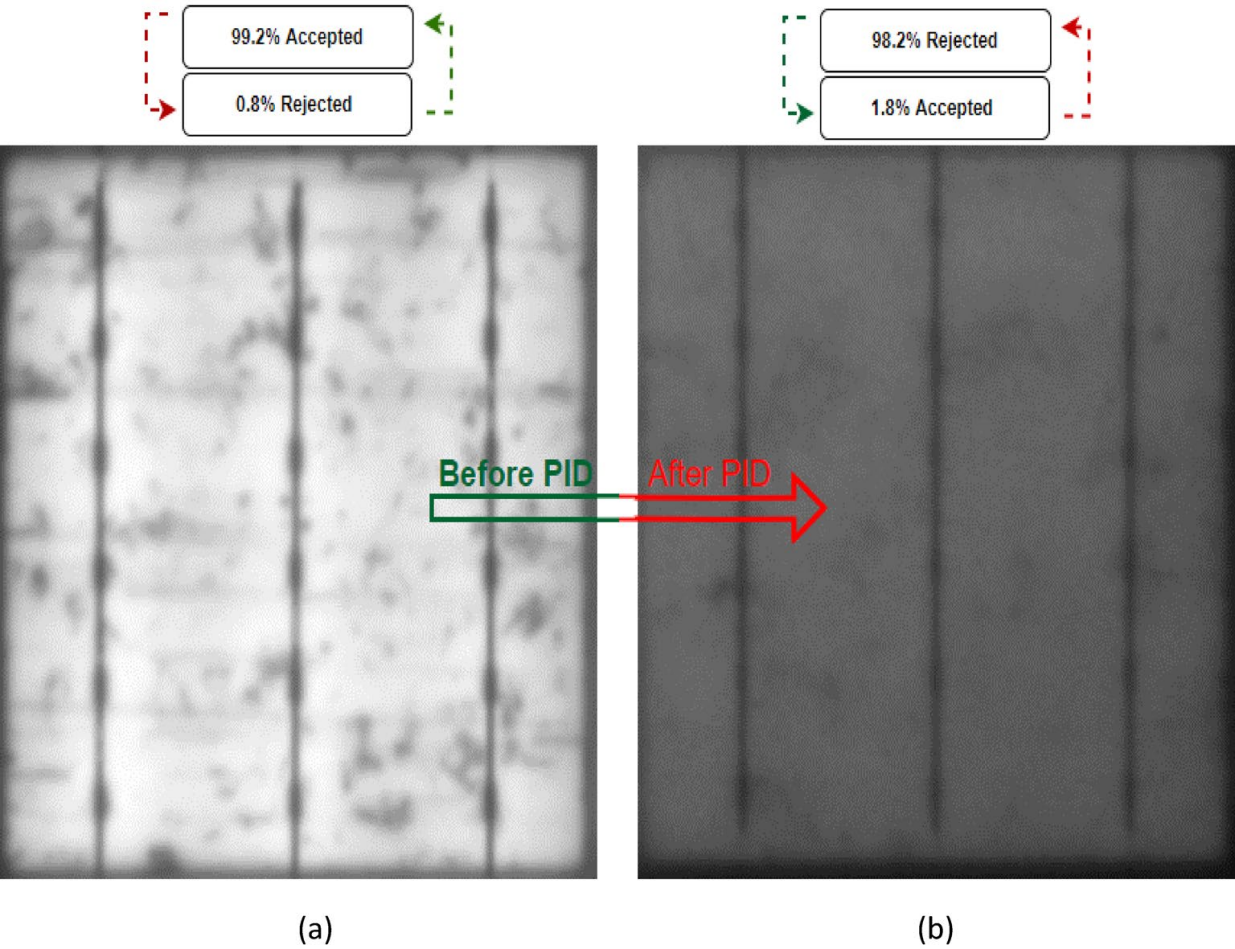
Examined solar cells with PID (case 5). (**a**) Before PID, (**b**) After PID.

There are many challenges associated with predicting whether a cell will be accepted or rejected, and one of the key challenges is predicting potential-induced degradation (PID). PID is a leading cause of module degradation and is caused by the high voltage generated between the encapsulants and the front glass surface, which is grounded through either the cell frame or the substructure^31^. As a result of this, we started to conduct testing on the solar cells before and after PID. The PID test was carried out while connecting the solar cells with − 1000 V for 96 h duration, and after we obtained the EL images before and after the PID, we put on the CNN system to test whether can predict the PID as accepted or rejected. After running the system, it was determined that (case 5) the acceptance rate of the solar cells before the PID was 99.2%, as shown in Fig. 9a. Despite the fact that the cells began to diminish after the PID occurred, this prediction resulted in a rejection of 98.2% for solar cells with a PID, as shown in Fig. 9b. These precise predictions demonstrate the effectiveness of the system in determining the acceptance rate of solar cells with and without a PID, making it a valuable tool for evaluating the condition of photovoltaic cells.

**Figure 10:**
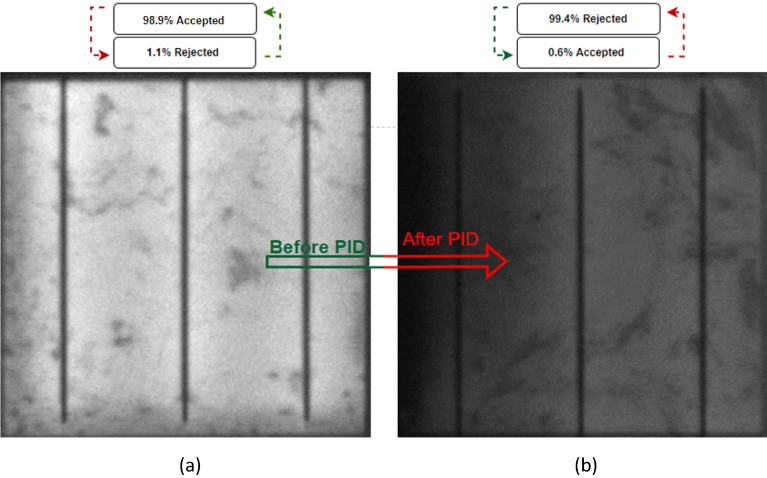
Examined solar cells with PID (case 6). (**a**) Before PID, (**b**) After PID.

For the purpose of further validation for the PID a further case (case 6) was tested on the system with the same characteristics as Case 5 but with a much more noticeable dimming specifically after the PID occurred as shown in Fig. 10a and b.

Upon running the system, it predicts that the system can effectively differentiate between the solar cells prior and subsequent to the PID by accepting the percentage of cells prior to the PID of 99.4%, while at the same time making accurate predictions for the cells with PID, with a rejection rate of 99.4%. This indicates a remarkable performance of the system and suggests that it is capable of distinguishing between cells that have undergone PID and those that have not, with a high degree of accuracy and precision.

In our study, we examined solar cells with shaded areas, which are commonly observed in silicon-based solar cells due to a phenomenon known as shunting. Shunting occurs during the manufacturing process and results in localized shaded regions on the solar cell's surface. By including shaded areas in our evaluation, we aimed to assess the effectiveness of our crack detection system in identifying and distinguishing between genuine cracks and these shunted regions.

**Figure 11:**
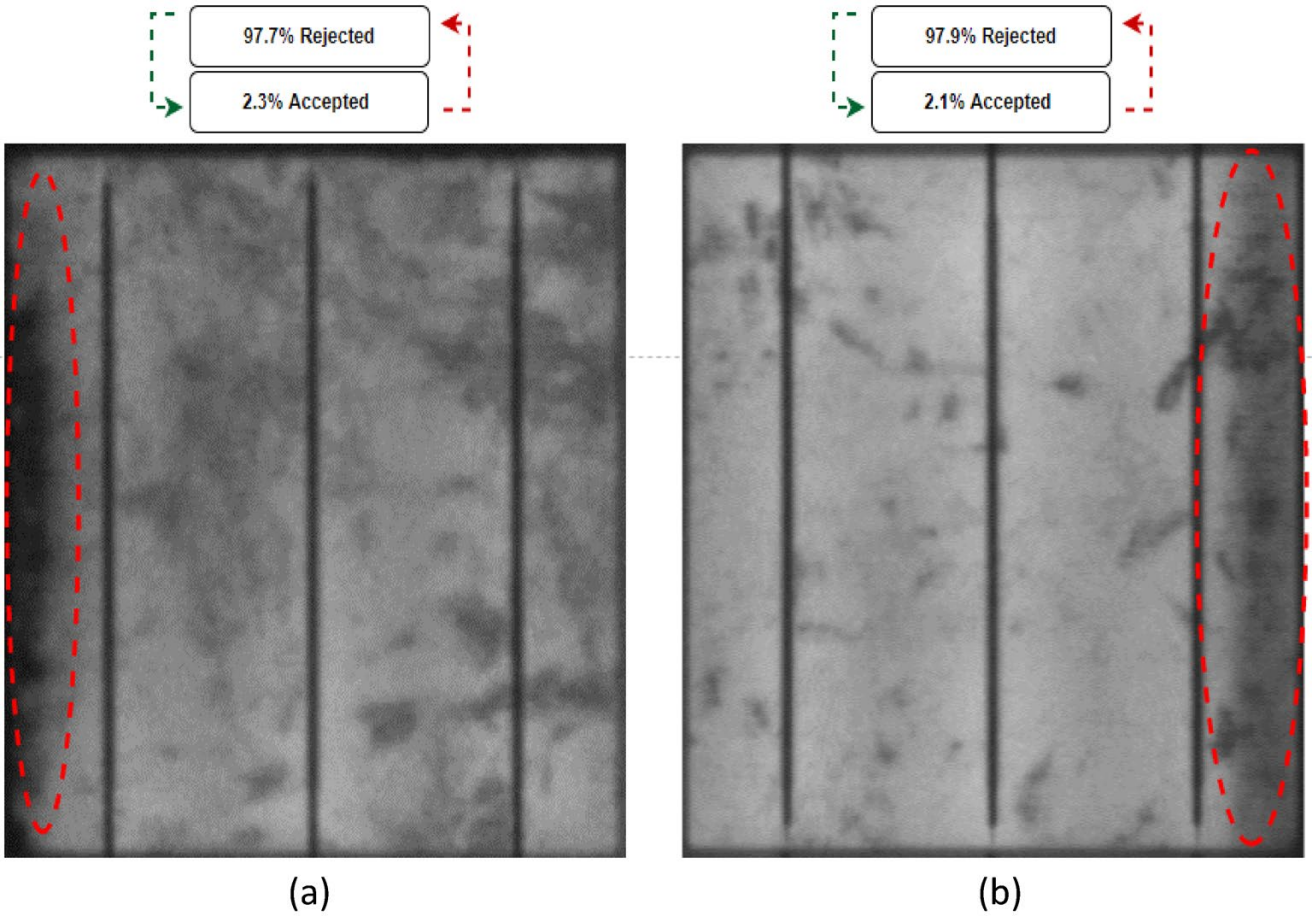
Examined solar cells with shaded area. (**a**) Case 7, (**b**) Case 8.

Solar cells with shaded areas (labelled in red dashed circles in Fig. 11) are especially challenging to predict whether they will be accepted or rejected. This is because the shade will cause an uneven distribution of current in the busbars. This would lead to stress in the cell and consequently higher temperatures. In the light of that, we took a look at two cases of solar cells with shaded areas, referred to as cases 7 and 8, and conducted the same processing test on them as on the others. Based on the results of the analysis, it was predicted that both cases would be rejected with 98.5% and 98.9%, respectively, as shown in Fig. 11a and b, which were accurate predictions given that the system has been trained to detect cells with shaded areas.

**Figure 12:**
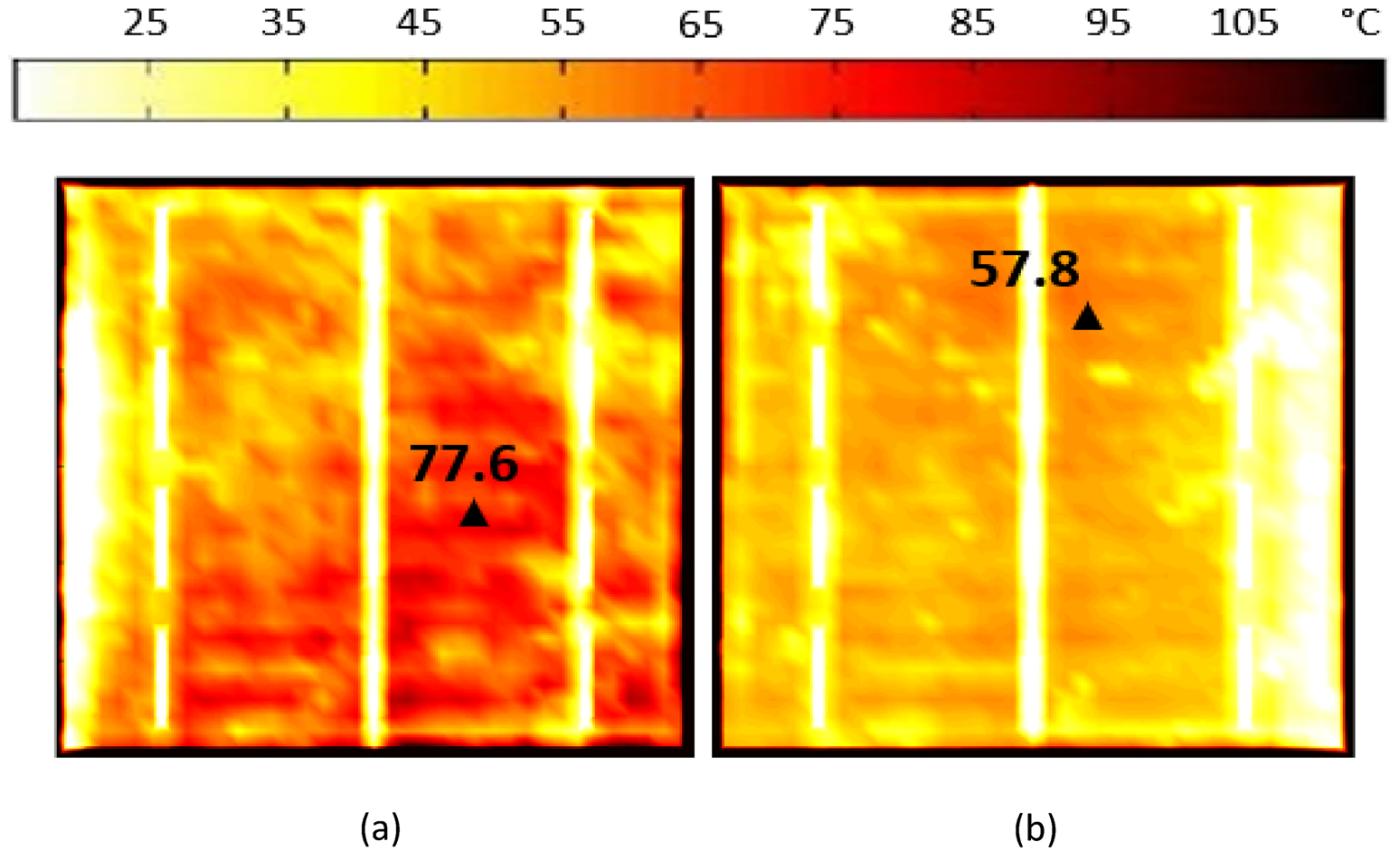
Thermal image of the examined solar cell with shaded area under STC Condition. (**a**) Case 7, (**b**) Case 8.

To provide further validation for the cases within the shaded area, we conducted thermal testing for both cases (case 7 and case 8) to put them on a solar simulator, under standard conditions, solar irradiance of 1000 W/m^2^ and cell temperature 25 °C are employed. Using thermal imaging, it is possible to determine whether a cell sample has hot spots. In both cases, after getting the thermal images, it was revealed that the temperature of the cells had increased to 77.6 °C and 57.8 °C respectively, as shown in Fig. 12a and b, which eventually led to power loss in each case.

**Figure 13:**
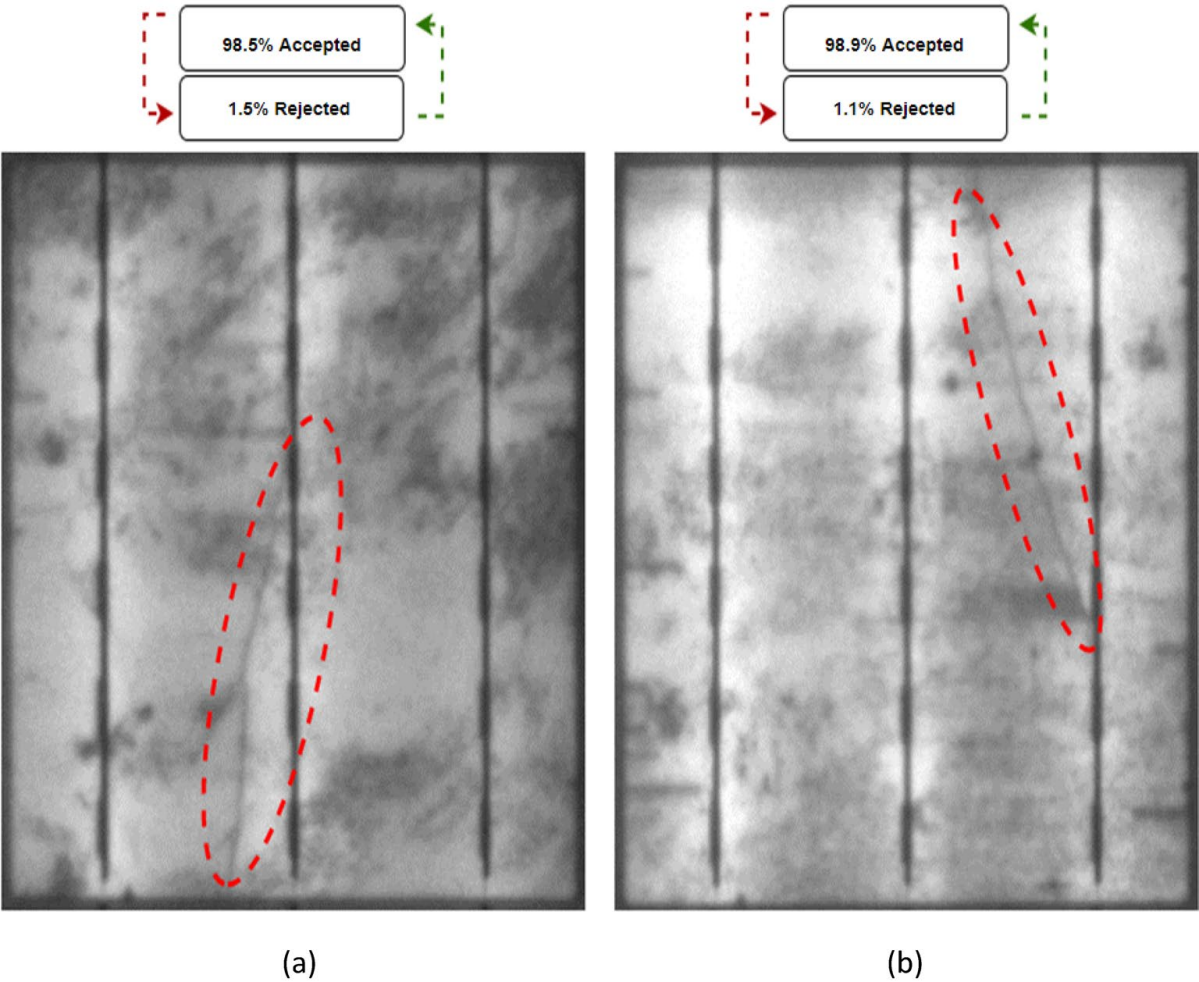
Examined solar cells with no major cracking. (**a**) Case 9, (**b**) Case 10.

A further factor to be considered when predicting the condition of solar cells is the presence of micro-cracks. Micro-cracks are not major cracks that can result in a big loss of power and can be classed as healthy cells as outlined in red dashed circles in Fig. 13a and b. Therefore, micro-cracks need to be taken into account when assessing the condition of solar cells, as they do not cause a significant decrease in energy output. As a result, we began examining two solar cells with micro-cracks (case 9 and 10), which prompted the system to identify them as accepted solar cells. In the end, the running system concluded that both cases were healthy, with a percentage acceptance of 99.5% and 99.9%, respectively. This indicates that it is a reliable prediction since it isn't a major crack and has minimal power loss.

**Figure 14:**
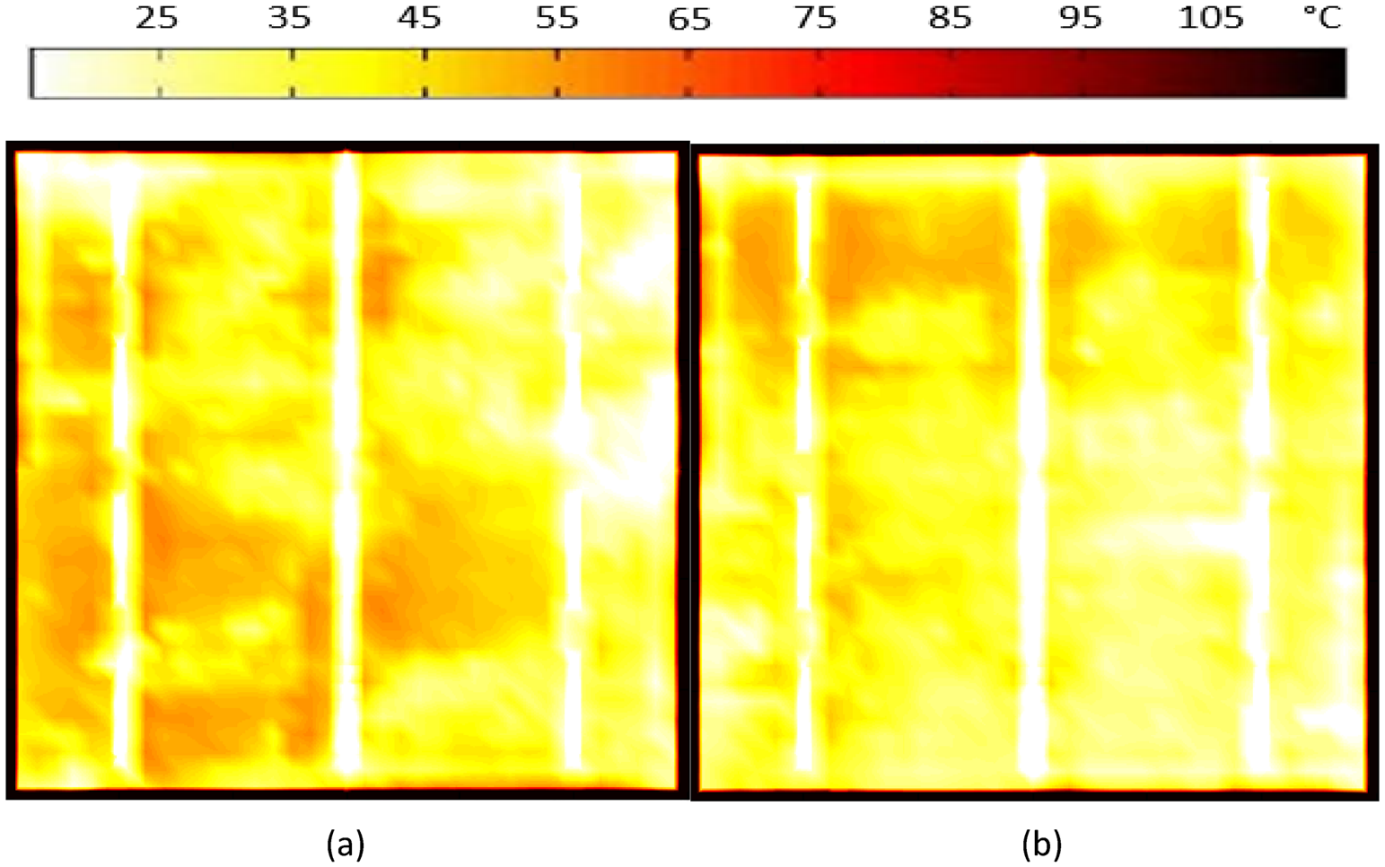
Thermal image of the examined solar cell with micro-cracks under STC Condition. (**a**) Case 9, (**b**) Case 10.

For the purpose of validating the results of the CNN system, thermal testing was conducted in both cases 9 and 10 by using the solar simulator with STC, with the same thermal imaging process that had been used in cases 7 and 8. As shown in Fig. 14a and b, the results of thermal testing indicated that a uniform distribution of heat across the surface of the solar cell was observed. Therefore, the surface temperature has been approximated to 25 °C, which is the standard temperature for testing. It is observed that the microcrack solar cells are not exposed to hotspots. It is also noteworthy that for the microcracks, there was no increase in the temperature of the cell, which confirms that microcracks do not change the temperature of the cell or cause a hot spot to develop in the cell.

## Comparative analysis

As a means of verifying the effectiveness of the proposed method, the obtained results have been compared with several well-developed CNN crack detection methods^26,28,32–34^ that are widely used in the industry. A summary of the comparison is given in Table 3.

**Table 3:** Comparison between our developed DSMP-CNN against several recently develop solar cell cracks detection algorithms^26,28,32–34^.

Ref	Year of study	Solar cell cracks detection description	Validation		
			Solar cell cracks	PID	Thermal test
^34^	2020	Pre-trained AlexNet-CNN: a CNN transfer learning crack detecting method based on pre-trained AlexNet network	✓	x	x
^28^	2021	BAFPN-CNN: is developed to accomplish multiscale feature fusion. This architecture, called bidirectional attention feature pyramid network (BAFPN), can make all layers of the pyramid share similar semantic features	✓	x	x
^26^	2021	In this study, pretrained models of VGG-16, VGG-19, Inception-v3, InceptionResNet50-v2, ResNet50-v2, and Xception are individually assessed before aggregating them using the ensemble method. Ensemble learning further increases the accuracy while reducing the risk of relying on a single model	✓	x	x
^33^	2022	PSO Pruner-CNN: The pruning problem of DCNN is formulated as a search problem, which is solved by particle swarm optimization (PSO) algorithm. To improve the quality of the pruning scheme, a tailored trick is considered that the automatic searching process with PSO algorithm is repeated for multiple rounds	✓	x	x
^32^	2022	Modified pre-trained AlexNet: pre-trained AlexNet architecture is modified to extract more detailed feature maps and a novel and efficient. multi-scale CNN model is proposed. Since low-level convolutions have small-sized filters, two convolutional branches are added to series-connected 3 × 3 convolutional layers	✓	x	x
This work	2023	DSMP-CNN: Dual Spin Max Pooling Convolutional Neural Network for Solar Cell Crack Detection. Through the development of new networks, we were able to assess the effectiveness of various architectures and improve the validation accuracy. Our approach of switching from mean pooling to maximum pooling, increasing the number of convolutional layers, and changing the pooling method led to significant improvements in validation accuracy	✓	✓	✓

Recent studies rely most heavily on transfer learning, which uses pre-trained networks instead of developing them from scratch by using different datasets. The most notable cases of transfer learning are^26,32,34^, which are based on AlexNet, VGG-16, VGG-19, Inception-v3, Inception ResNet50-v2 and ResNet50-v2. Although the cases weren't developed from scratch, the pre-trained network was tweaked a bit by^32^. Interestingly, all these studies are competent to detect cracks, but not PIDs and shaded areas. Additionally, the results have not been validated with thermal testing. Furthermore, these studies do not discuss the potential of transfer learning to detect PIDs and shaded areas, or the possible benefit of thermal testing in validating the results.

Based on^28,33^ developed CNN architectures, bidirectional attention feature pyramid network (BAFPN) and swarm optimization (PSO), both networks are able to detect cracks as efficiently as previous networks. In addition to their reluctance to detect PIDs and shaded areas, these networks have not been validated with thermal testing, and tending to require extensive data to train.

As a result of this study, four different architectures were developed from scratch, and we were able to assess the effectiveness and accuracy of these architectures as well as to improve the reliability of validations. We have validated the results of this network with thermal testing in order to be able to rely on it in PV assembly units to identify cracks, micro cracks, PID and shaded areas. Using this network, PV assembly units will be able to classify various solar cells in the most straightforward manner possible.

While this paper presents significant advantages, there are certain limitations to be acknowledged in this study. Firstly, the evaluation of the proposed system was conducted using a specific dataset, which might not fully capture the diversity of real-world scenarios involving various types of solar cells. For instance, solar cells with different busbar configurations or manufacturing variations were not extensively explored. Therefore, the generalizability of the findings to these specific cases may be limited.

Secondly, the study relied on EL images obtained from indoor environments and a solar cell production line. While these settings are relevant to the intended application, it is important to recognize that imaging conditions can vary in different scenarios. Factors such as lighting conditions, camera specifications, and imaging setups may differ when examining solar cells in outdoor environments or using different EL detection equipment. Hence, the transferability of the proposed system to these alternative imaging conditions may require further investigation and adaptation.

To address these limitations in future research, it is recommended to:

- Expand the dataset to encompass a broader range of solar cell types, including variations in busbar configurations and manufacturing processes, to improve the representativeness of the evaluation.
- Incorporate EL images captured in diverse imaging conditions, such as outdoor environments or using different EL detection equipment, to assess the system's performance under various scenarios.
- Consider conducting comparative studies or benchmarking against existing crack detection methods using multiple datasets to establish the robustness and effectiveness of the proposed system across different settings.
- Collaborate with industry partners or research institutions to access a wider range of solar cell samples and imaging conditions, ensuring the findings are applicable to real-world scenarios.
- Conduct sensitivity analyses or perform experiments to evaluate the system's performance under controlled variations in lighting conditions, camera settings, or other imaging parameters to assess its robustness and adaptability.

By addressing these limitations, future research can enhance the applicability and reliability of the crack detection system for a broader range of solar cell types and imaging conditions.

## Conclusions

A novel solar cell crack detection system for application in PV assembly units was developed and presented in this article. A proposed network incorporates four different CNN architectures with varying validation accuracy to detect cracks, microcracks, PIDs, and shaded areas, supported by thermal testing to validate the results. In this method, the system examines the EL image of the solar cell and identifies whether it predicts the solar cell to be accepted or rejected with precise. Moreover, the system utilizes a set of features to reduce the false-positive rate and increase accuracy of the crack detection process using a dual spin max pooling CNN architecture.

The proposed CNN system was tested on various solar cells to determine their acceptance/rejection status. The system was found to be highly accurate in determining the condition of solar cells based on the presence and size of black spots, cracks, PID, and shaded areas. The system achieved a high degree of accuracy, with an acceptance rate of up to 99.5% and rejection rate of up to 99.1% in different cases. Moreover, the system was validated with thermal testing using cases with real ranges, such as shaded areas and micro-cracks, which were predicted with high degrees of accuracy by the system. The results indicate that the proposed system is a valuable tool for evaluating the condition of photovoltaic cells and can lead to improved efficiency. Moreover, the study uncovered a superior performance of the proposed CNN model in comparison with previous studies in the detection of cracks, micro-cracks, PIDs, and shaded areas on solar cells.

This research is important to the PV industry and PV solar cell crack detection automation for several reasons:

- Accurate crack detection: The proposed DSMP-CNN system can accurately detect cracks, microcracks, PIDs, and shaded areas in photovoltaic cells, which can lead to improved efficiency and reliability of the cells.
- High accuracy: The system was tested on various solar cells and achieved a high degree of accuracy, with an acceptance rate of up to 99.5% and rejection rate of up to 99.1% in different cases.
- Improved efficiency: By automating the crack detection process, the proposed system can reduce manual inspection time and errors, leading to improved efficiency in the PV industry.
- Real-time detection: The system can quickly examine the EL image of the solar cell and predict its acceptance or rejection status in real-time, which can save time and resources for PV companies.

## Supplementary Information

Supplementary Figures.

## Data Availability

The dataset generated and analysed in this study may be available from the corresponding author (S.H.) on reasonable request.
