# Supplementary material for: Dual spin max pooling convolutional neural network for solar cell crack detection
Source: Sci Rep. 2023 Jul 9;13:11099. doi: 10.1038/s41598-023-38177-8 (PMC10330187; doi:10.1038/s41598-023-38177-8)
Supplement: Supplementary file 1 — Supplementary Figures. [file 41598_2023_38177_MOESM1_ESM.docx]

**Supporting Information**

**Dual Spin Max Pooling Convolutional Neural Network for Solar Cell Crack Detection**

Sharmarke Hassan*, Mahmoud Dhimish

Photovoltaics Laboratory, School of Physics, Engineering and Technology, University of York, York YO10 5DD, United Kingdom

Corresponding Author: [smh600@york.ac.uk](mailto:smh600@york.ac.uk)


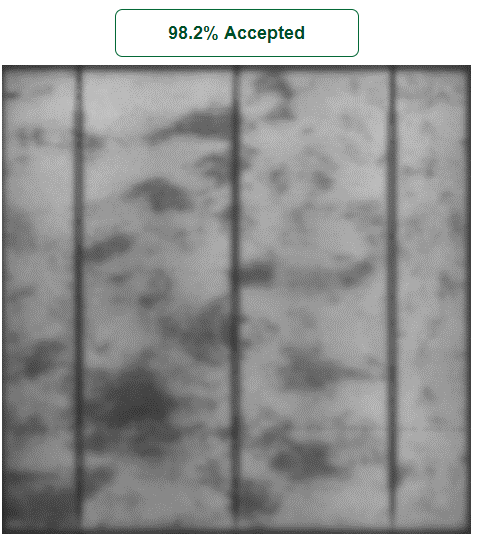

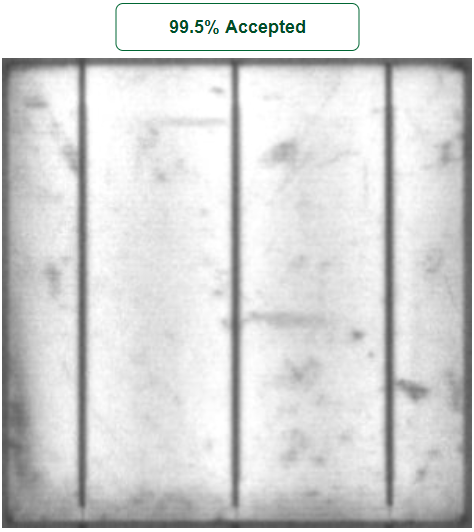

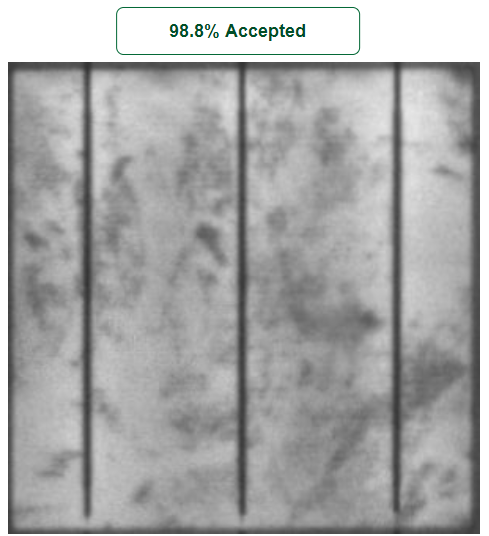


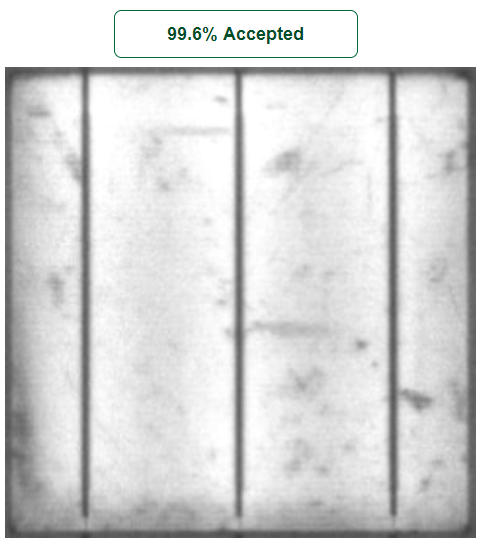

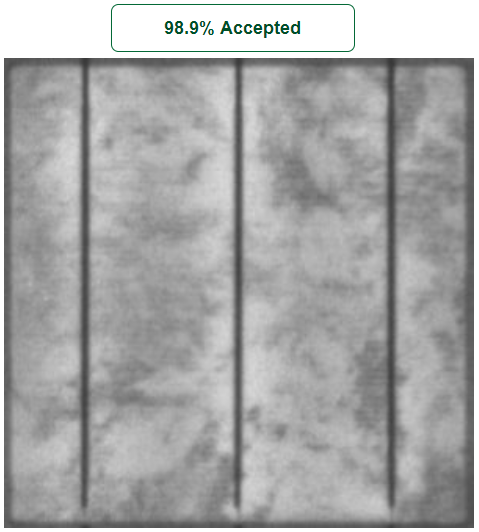

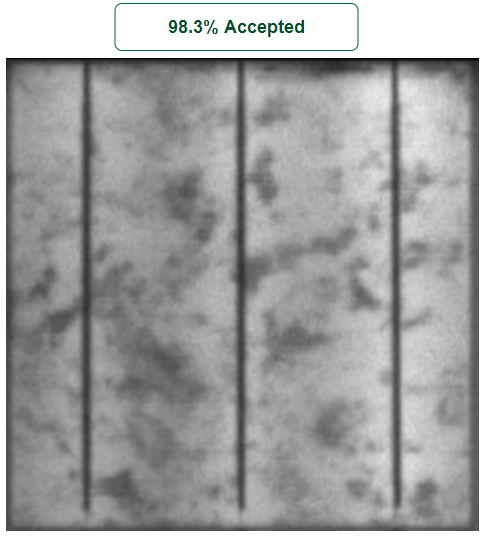


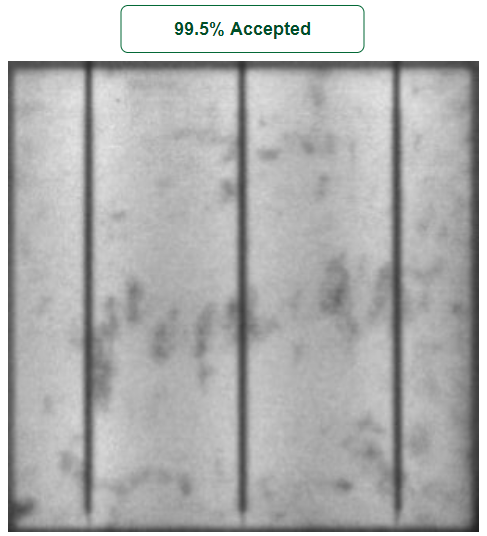

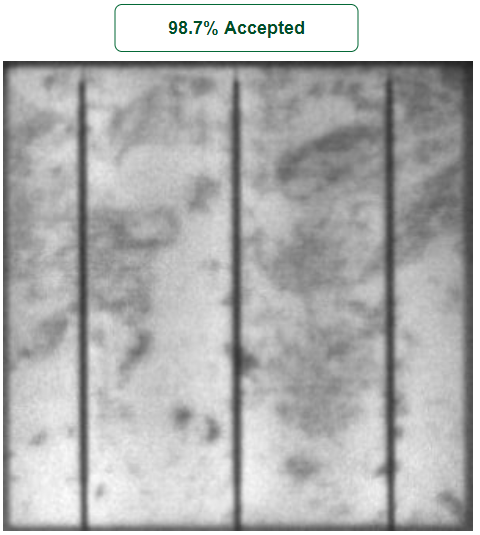

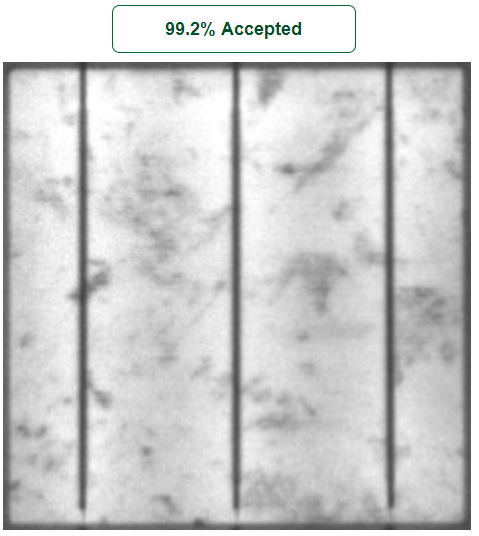

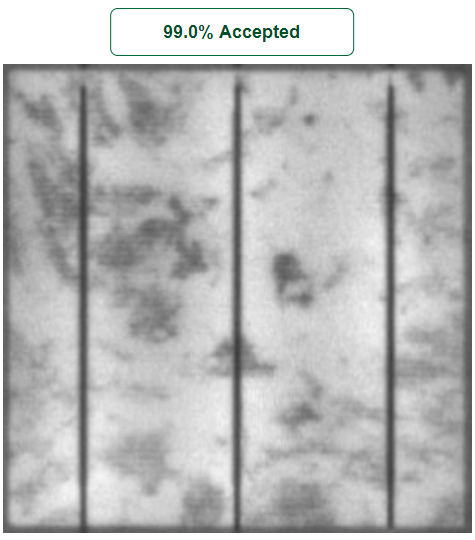

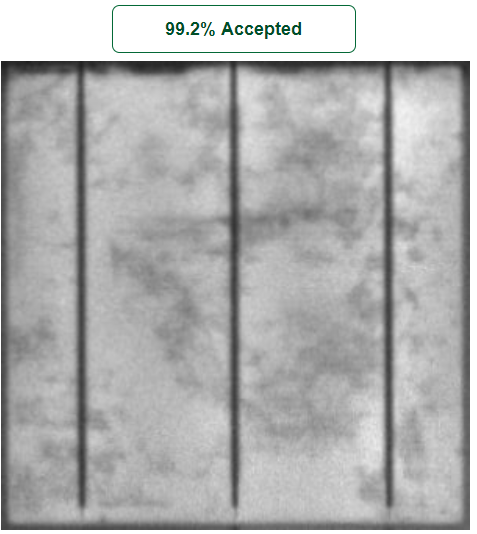

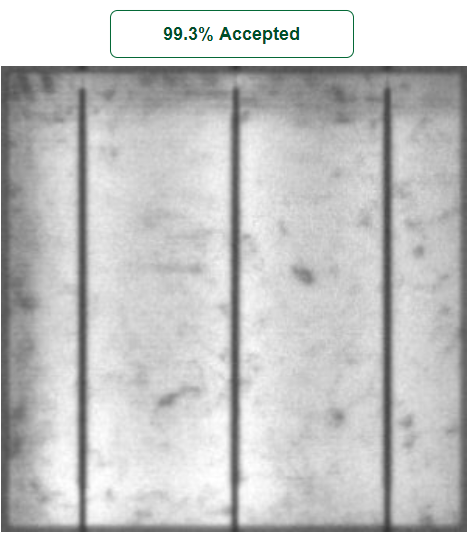


**Figure S1**: Further examples of 12 examined healthy solar cells with predicted acceptance rates.


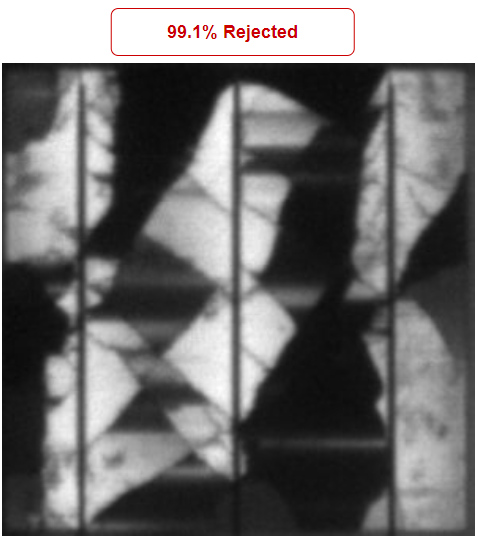

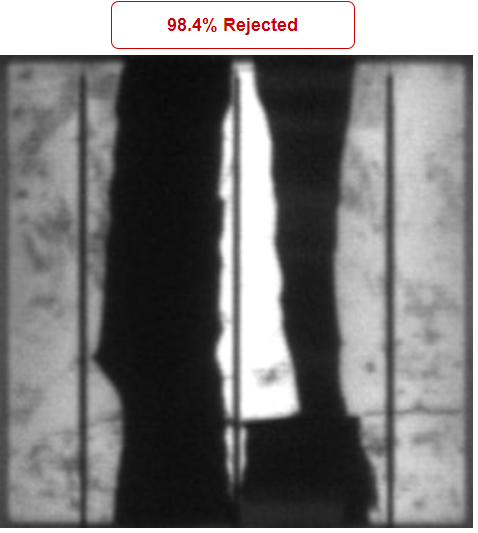

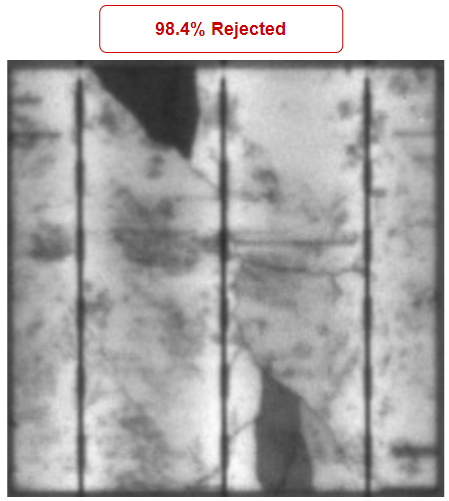

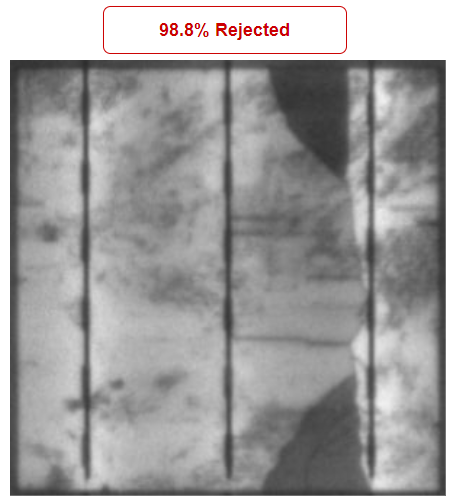

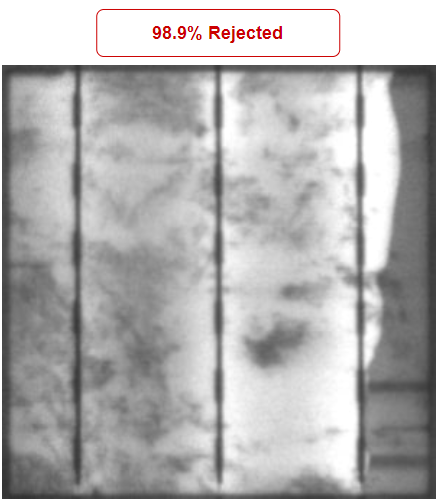

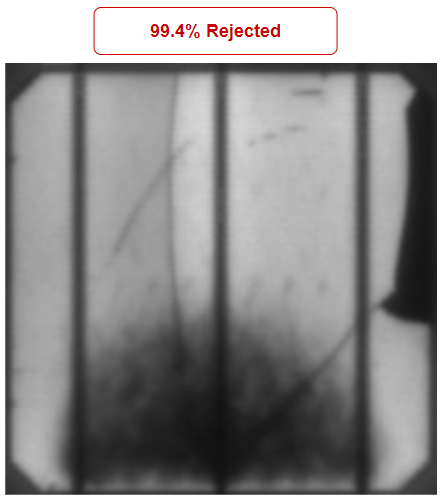

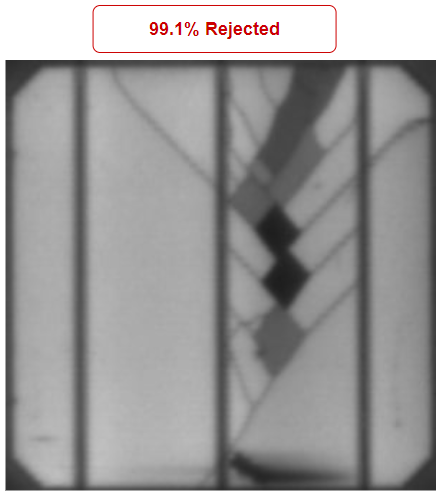

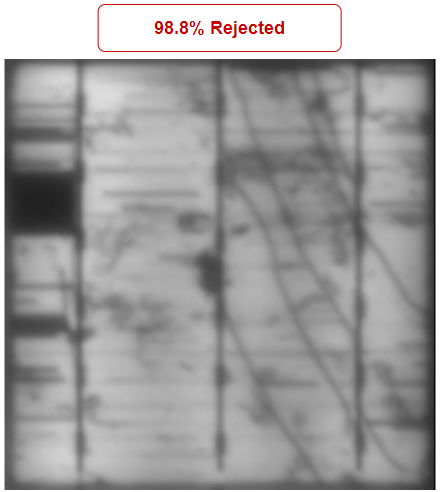

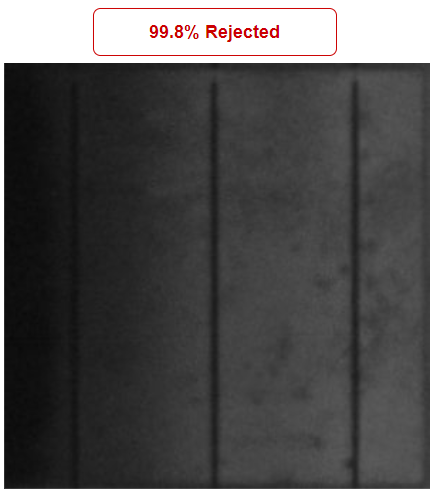


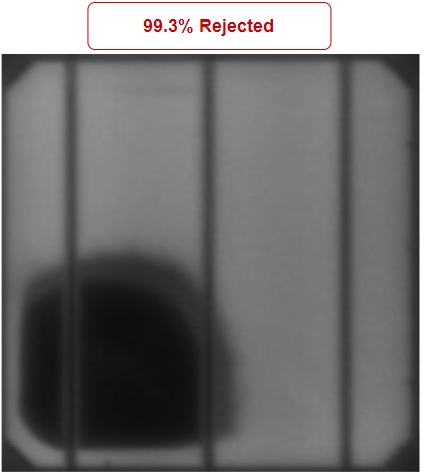

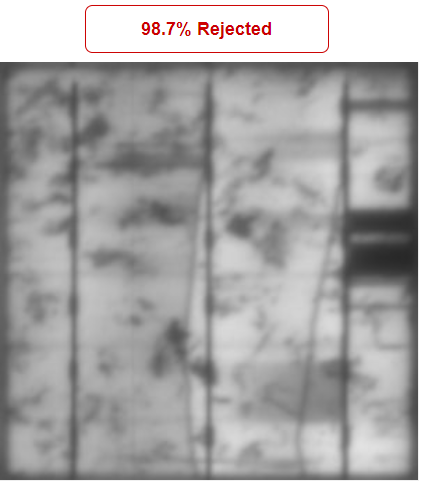

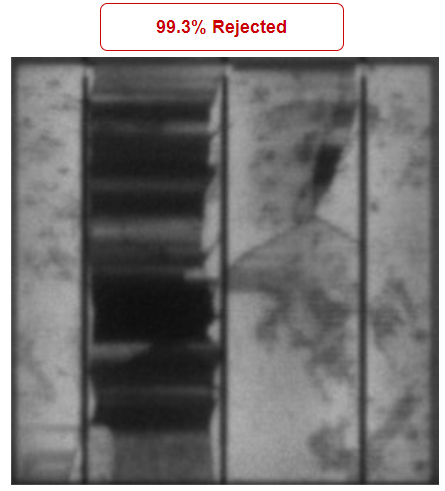


**Figure S2**: Further examples of 12 examined cracked solar cells with predicted rejection rates.
